# A Convenient All-Cell Optical Imaging Method Compatible with Serial SEM for Brain Mapping

**DOI:** 10.3390/brainsci13050711

**Published:** 2023-04-24

**Authors:** Tianyi Wang, Peiyao Shi, Dingsan Luo, Jun Guo, Hui Liu, Jinyun Yuan, Haiqun Jin, Xiaolong Wu, Yueyi Zhang, Zhiwei Xiong, Jinlong Zhu, Renjie Zhou, Ruobing Zhang

**Affiliations:** 1 School of Biomedical Engineering (Suzhou), Division of Life Sciences and Medicine, University of Science and Technology of China, Suzhou 215163, China; 2 Jiangsu Key Laboratory of Medical Optics, Suzhou Institute of Biomedical Engineering and Technology, Chinese Academy of Sciences, Suzhou 215163, China; 3 Institute of Artificial Intelligence, Hefei Comprehensive National Science Center, Hefei 230088, China; 4 State Key Laboratory of Digital Manufacturing Equipment and Technology, Huazhong University of Science and Technology, Wuhan 430074, China; 5 Department of Biomedical Engineering, The Chinese University of Hong Kong, Hong Kong, China

**Keywords:** optical multilayer interference tomography, brain mapping, correlative light and electron microscopy, cell classification

## Abstract

The mammalian brain, with its complexity and intricacy, poses significant challenges for researchers aiming to understand its inner workings. Optical multilayer interference tomography (OMLIT) is a novel, promising imaging technique that enables the mapping and reconstruction of mesoscale all-cell brain atlases and is seamlessly compatible with tape-based serial scanning electron microscopy (SEM) for microscale mapping in the same tissue. However, currently, OMLIT suffers from imperfect coatings, leading to background noise and image contamination. In this study, we introduced a new imaging configuration using carbon spraying to eliminate the tape-coating step, resulting in reduced noise and enhanced imaging quality. We demonstrated the improved imaging quality and validated its applicability through a correlative light–electron imaging workflow. Our method successfully reconstructed all cells and vasculature within a large OMLIT dataset, enabling basic morphological classification and analysis. We also show that this approach can perform effectively on thicker sections, extending its applicability to sub-micron scale slices, saving sample preparation and imaging time, and increasing imaging throughput. Consequently, this method emerges as a promising candidate for high-speed, high-throughput brain tissue reconstruction and analysis. Our findings open new avenues for exploring the structure and function of the brain using OMLIT images.

## 1. Introduction

Brain mapping tools are vital for investigating the relationship between brain structure, higher cognitive functions, and the underlying mechanisms of various neurological diseases [1]. Although the concept of brain maps was recognized over a century ago [2], a majority of the fine cellular structures within the nervous system remain unexplored due to the large volume and complexity of mammalian brain tissue [3]. Understanding the structure and mechanisms of entire neural networks, where multiple neurons are interconnected and work together, is essential for deciphering the principles governing brain functions [1,4,5], advancing our knowledge of the brain, and improving the diagnosis and treatment of neurological disorders [6].

Various volume electron microscopy techniques have been developed to image and reconstruct brain maps, thereby allowing the identification of the network of synaptic connections between neurons at high resolution [7,8,9]. Serial block-face electron microscopy (SBEM) and focused ion beam scanning electron microscopy (FIB-SEM) have been used to image the surface of a tissue block, followed by removing the top surface to expose a new surface for imaging [10,11,12,13,14]. In recent years, the femtosecond-laser-enabled FIB SEM (TriBeam) technology has been optimized for use in damage-sensitive materials, such as biological tissues, achieving rapid serial sectioning and imaging with material removal rates significantly higher than traditional Ga FIB [15,16,17,18]. Automated tape-collecting ultramicrotome scanning electron microscopy (ATUM-SEM) has been developed to serially image the sections by collecting ultrathin sections on tapes [19,20,21]. These imaging methods, when combined with manual or automated image segmentation and recognition, enable the creation of detailed and precise morphological and distribution maps of each neuron within the field of view of brain neural networks [22,23]. Additionally, they reveal synaptic-level connections [24], ultimately allowing for the reconstruction of comprehensive and intricate micro-connection maps of complex local neural networks [25,26]. However, due to low imaging throughput and a limited field of view, the time required for mammalian brain-scale reconstruction using electron microscopy remains exceedingly long [3], even when employing multi-electron beam SEM technology that is tens of times faster [27,28,29,30]. Additionally, the substantial computational resources and time costs needed for fusing, stitching, registering, or segmenting the vast datasets generated using electron microscopy present a significant challenge, and the hardware costs remain high.

Several fluorescence-based optical microscopy methods have been proposed for brain mapping, including the fluorescence-specific labeling-based fluorescence micro-optical sectioning tomography (fMOST) developed in 2013 [31,32,33], the two-photon microscopy technique reported in 2016 [34], and the volumetric imaging with synchronized on-the-fly-scan and readout (VISoR) system established in 2019 [35]. However, these methods can only image sparsely labelled cells and cannot map the morphology and projections of all neurons or enable brain mapping within the same sample. Label-free optical imaging techniques including functional photoacoustic microscopy [36,37], optical coherence tomography [38], and quantitative phase microscopy [39] have also been explored. While these methods have the potential to map every neuron in the brain, they typically suffer from low spatial resolution and limited imaging depth.

Clearly, integrating low-resolution, large-scale tomographic imaging with ultra-high-resolution, small-volume continuous electron microscopy is crucial for multiscale brain atlas analysis. However, current correlative light-electron imaging techniques mainly focus on merging the specific labeling and localization capabilities of fluorescence microscopy for cell types, proteins, or nucleic acids with the spatial resolution advantages of electron microscopy. This approach reveals the precise localization of fluorescently labeled proteins within the electron microscopy context [40,41,42,43,44] but is unsuitable for brain atlas imaging demands that require combining the extensive field of view of light microscopy with the high resolution of electron microscopy. The previously mentioned optical tomographic brain atlas imaging techniques [31,32,33,34,35] are also incompatible with electron microscopy samples, preventing their direct integration with continuous electron microscopy brain atlas imaging. Recently, X-ray microtomography has been employed for the quantitative analysis of mesoscale neuroanatomy [45], providing valuable guidance for subsequent scanning electron microscopy (SEM) imaging. However, the images obtained through this technique can only guide the selection of regions of interest (ROI) and navigation within ATUM-SEM when projected onto the actual plane where the ultramicrotome sections the specimen. This process necessitates the execution of intricate graphical operations, potentially leading to inaccuracies, and the associated system entails considerable expense.

Optical multilayer interference tomography (OMLIT) was recently reported [46] and showed its capability as an imaging technique for generating mesoscale and microscale brain maps within a single sample. However, the method was easily limited by imperfect coating processes, which yielded metal layers with structured and scratched surfaces, causing confusion and contamination in the images of cells.

To address these limitations, we introduce a novel coating-free OMLIT protocol that is correlated with a tape-based serial SEM imaging workflow. Our method combines large-scale tomography with a high-resolution serial imaging method, achieving low-cost and high-throughput mesoscale brain mapping. Compared to current configurations of OMLIT [46], we omit the step of coating on the collection tape to address issues such as uneven surfaces resulting from non-uniform atomic deposition, fractures caused by poor ductility of metal, and peeling and warping due to inadequate bonding of metal coating, as well as problems arising from contamination and scratching of the metal coating surface. The removal of this step resulted in fewer artificial defects as well as reduced costs since there is no need for an expensive coating instrument and metal targets. We addressed the need for the conductivity of the sample surface by spraying carbon on the surface of the sample sections.

In this study, we have optimized the results of existing OMLIT and, after evaluating the imaging quality, validated the feasibility of this coating-free OMLIT–SEM correlation imaging protocol in achieving higher image contrast with less noise. We have also demonstrated the applications of this method in a large-scale, three-dimensional (3D) reconstruction of large-volume mouse cortex samples and cell classification. When optimizing the contrast and resolution of this method, we have also explored other influencing factors, such as thickness of sample sections and bandwidth of light sources. Our proposed coating-free OMLIT–SEM imaging method has the potential to enable large-scale, high-resolution brain mapping studies at a lower cost and with higher throughput, which will greatly facilitate our understanding of brain structure and function in the future.

## 2. Materials and Methods

**Figure 1:**
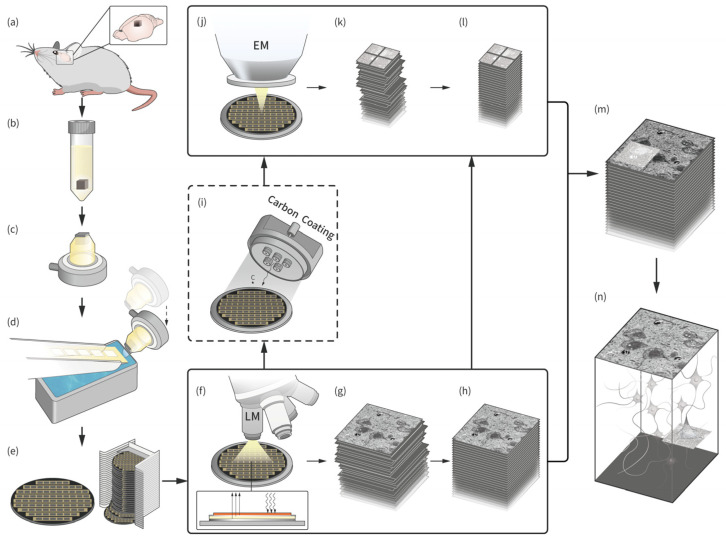
Steps taken to generate light microscopy (LM) and electron microscopy (EM) datasets. (**a**) Obtaining cortex tissue sample from mouse brain. (**b**) The tissue block stained with heavy metals is immersed in resin for preservation and fixation. (**c**) The resin block is trimmed to expose the sample surface. (**d**) The sample block is cut into serial ultrathin sections and collected on a tape. (**e**) Tapes with collected sections are cut, placed on conductive carbon tapes covering a silicon wafer, and used to generate wafer libraries for a sample, enabling multiple imaging sessions. (**f**–**h**) Optical imaging and alignment to generate LM dataset. (**i**) Optional step: if the tape collecting the sample is not conductive, the surface of the wafer should be coated with carbon after optical imaging. (**j**–**l**) Electron microscope imaging, stitching, and alignment to generate EM dataset. (**m**) Co-alignment of LM and EM datasets. (**n**) Three-dimensional reconstruction result.

An overview of the sequence of data generation steps in this study is presented in Figure 1. Brain tissue from mice was obtained and prepared for electron microscopy, which involved fixation, staining, dehydration, and embedding of the tissue blocks in resin. Ultrathin sections of the tissue blocks were then cut using an ultramicrotome and collected on polycarbonate tapes with or without metal coating. These tapes were split and attached to the silicon wafer and imaged first with an optical microscope and then with an electron microscope. If the tape surface was not coated with a conductive metal, it was necessary to spray the surface with carbon before electron microscopy imaging could be performed. The resulting datasets were stitched and aligned with ImageJ or a special algorithm, and manually segmented to complete a 3D reconstruction.

### 2.1. Optical Model Simulation

In order to investigate the optimal OMLIT configuration for the best imaging results, we employed a matrix laboratory (MATLAB) program that describes the reflection and interference phenomena within a multilayer structure for the optical model [46], simulating the light intensity reflected from the cell and its surrounding tissue. During the simulation, it was assumed that both the incident and exit media were lossless dielectrics, and that all layers within the multilayer structure exhibited nonmagnetic properties. Parameters such as angle of incidence, polarization state, the wavelength of the incident light, refractive index, extinction coefficient, and thickness of each layer were input to calculate the reflectance R [47]. Figure 1f shows a schematic of the multilayer structure. In a top-down order, there are four thin-film layers above the silicon wafer, sequentially comprising: an ultrathin section, a metallic coating (not present in the no-coating scheme), a polymorphic tape, and a conductive carbon tape. Refractive indices (n) and extinction coefficients (k) for various metals, resins, staining agents, brain tissue, and polycarbonate membranes were obtained from the existing literature or measured using an ellipsometer.

### 2.2. Fabrication of Metal-Coated Tapes

In order to reduce the background noise of the multilayer structure, we selected the best-performing D50 tape (a 50-micron thick Panlite PC film, Teijin) and experimented on it with various coating materials. To adjust the reflectivity, a thin layer of silver (Ag), chromium (Cr), or copper (Cu) was deposited onto the uppermost surface of the tape. Metallic material deposition was achieved using the magnetron sputtering (Magnetron Sputtering Deposition System, Yujie) method, resulting in a high-density, uniformly distributed thin-film coating with strong adhesion between the metal layer and the underlying tape. To optimize the deposition process, the substrate tape was set at a distance of 80 mm from the metal target, employing direct current (DC) power and 1.0 Pa pressure, with the pressure regulated by 99.9999% pure argon gas. An electric winding device was responsible for collecting the metal-deposited tape. After the deposition process was completed, the chamber’s temperature gradually decreased to room temperature under high vacuum conditions, minimizing internal stress [48]. Coating thicknesses were assessed using a stylus profiler (DektakXT, Bruker, Billerica, MA, USA), an atomic force microscope (AFM, Dimension Icon, Bruker), and a scanning electron microscope (GeminiSEM 300, Zeiss, Oberkochen, Germany) for calibration curve fitting. By adjusting the tape’s translation speed, a range of coating thicknesses was achieved, including 30, 50, 70, 100, and 150 nm.

### 2.3. Sample Preparation

Experiments were carried out with 5-week-old male mice (*n* = 2) following the institutional Ethics Committee (protocol ID 2022-A55). Specifically, two 5-week-old male Shank3B^+/−^ mice were anesthetized using a combination of Zoletil (55 mg/kg) and xylazine (5 mg/kg). They then underwent transcardial perfusion, with an initial infusion of 30 mL of artificial cerebrospinal fluid, followed by an injection of 30 mL of fixative solution to preserve the fine structure of the biological tissue [46]. The brain was carefully removed from the skull, and a cortical tissue block approximately 2 × 2 × 2 mm^3^ in size was excised. The tissue was then subjected to chemical fixation, sequential heavy metal staining, dehydration, and resin embedding, in accordance with a previously established protocol [49].

After the resin solidification, we trimmed the sample and proceeded to section and collect a series of ultrathin slices with varying thicknesses from the sample blocks, placing them on selectively coated tapes as previously described (UC7, Leica, Wetzlar, Hessen, Germany). We used an automated tape-collecting ultramicrotome (ATUM) (Figure 1d) [21] to collect sample sections of 30, 40, 50, 60, 70, 80, 90, and 100 nm thicknesses on various coatings, including chromium, copper, and silver-coated D50, as well as uncoated D50, to investigate the influence of section thickness on image contrast. Additionally, we collected 60-nm-thick sample sections on the surface of all tapes with different coating thicknesses to examine the effect of coating thickness and composition on imaging quality. After section collection, the substrate tapes were placed on top of a black double-sided adhesive carbon conductive tape (Ted Pella) that covered a silicon wafer. To perform large-scale reconstruction, we collected a total of 1657 cortex sample sections (1 × 0.83 mm^2^, 55 nm thickness), all of which underwent poststaining with uranium acetate and lead citrate to enhance image contrast before optical imaging.

In particular, uncoated samples requiring electron microscopic imaging were surface-carbon-sprayed with a thickness of 5.7 nm for 20 pulses with a high-vacuum sputter coater (ACE600, Leica) after the light microscopic imaging is completed.

### 2.4. Imaging

We collected a total of 1657 cortex sample section images using an upright reflected light microscope (VS200, Olympus, Tokyo, Japan), which was equipped with four monochromatic light-emitting diode (LED) illumination devices (FluoCa Fc906, Olympus), a 2× objective (Plan N 2 × 0.06, Olympus), a 20× objective (MplanApo N 20 × 0.60, Olympus), and a charge-coupled device (CCD) camera (VS-304M, Olympus) to obtain continuous optical images. Based on the density and morphology of neurons and blood vessels in the OMLIT images, we deliberately selected an ROI and subsequently imaged it using a multi-beam scanning electron microscope (MultiSEM 505, Zeiss) under specific imaging conditions. These conditions included a single-beam electron current of 570 pA, an accelerating voltage of 1.5 kV, a working distance of 1.4 mm, a scan speed of 1.25 MHz, a dwell time of 0.8 μs, and a point scanning mode. The signal used was secondary electrons (SE2). The resulting sequential images had a lateral pixel resolution of 4 nm.

### 2.5. Data Analysis

The images were normalized, and equivalent areas were selected within both the cell body and surrounding tissue to calculate the average brightness, which was used to determine the contrast [46]. For each OMLIT configuration with specific coating thickness and section thickness, 50 pairs of cells and surrounding tissue areas were evaluated to calculate the average contrast. The mouse cortex light microscopy (LM) dataset was aligned using the Register Virtual Stack Slices tool in ImageJ, while the electron microscopy (EM) dataset was aligned using the mb_aligner algorithm based on the elastic algorithm [50,51,52]. The correlative light and electron microscopy datasets were aligned with each other using the Linear Stack Alignment with SIFT (Scale-Invariant Feature Transform) plugin in ImageJ. The 3D pattern of the mouse cortex was manually segmented using the custom volume annotation and segmentation tool (VAST) tool [53], and cell analysis was performed on this dataset.

## 3. Results

### 3.1. Simulation Results

**Figure 2:**
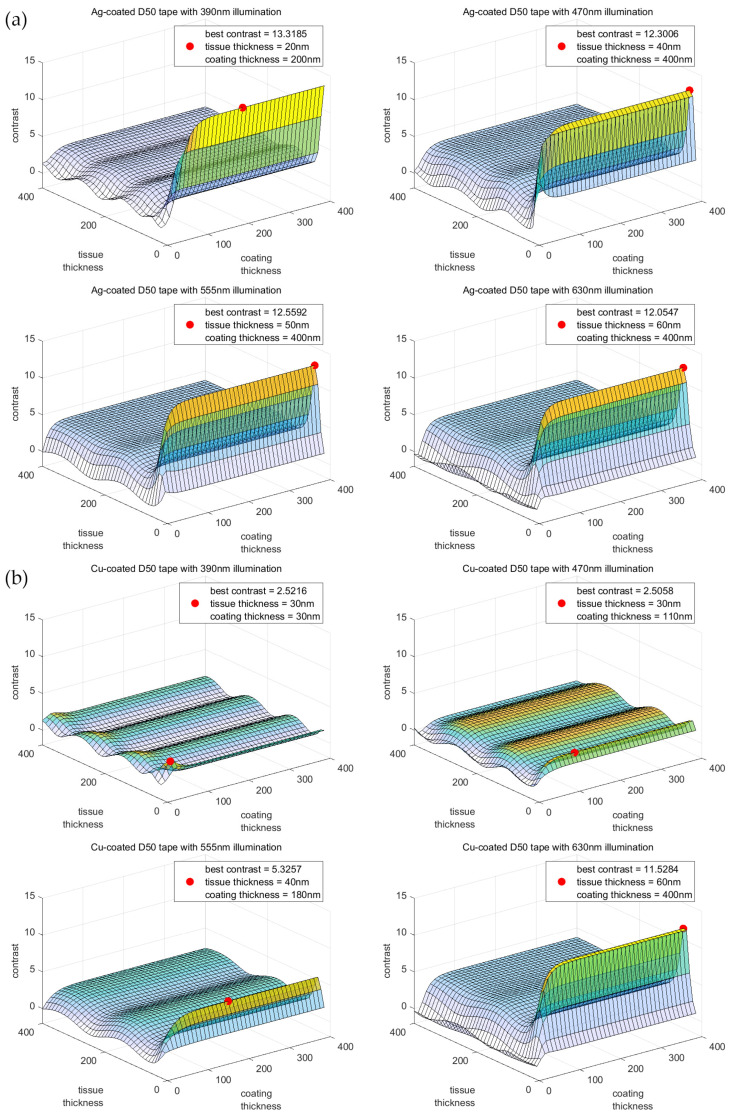
Contrast simulation results for (**a**) silver and (**b**) copper coating at different coating thicknesses and slice thicknesses on a D50 polycarbonate film substrate at different incident wavelengths. The red dots denote the locations of maximal contrast, with corresponding contrast conditions indicated in the legends.

To explore the effect of monochromatic light sources and different metallic coating materials on contrast, we optimized the original MATLAB model [46,47,54] of a white light multilayer film interference to a monochromatic light model based on the four LED light sources equipped with an Olympus VS200 microscope. We then predicted the contrast of multiple material configurations, with the contrast simulation results under different light sources shown in Figure 2 and Figure S1.

We observed that the contrast fluctuated periodically as the slice thickness increased, and the length of the period was proportional to the wavelength of the incident light. This finding suggests that our method can modulate the imaging contrast of thick slices to achieve superior image quality even when thicker sections are used as samples. The optimal contrast and corresponding conditions simulated for all configurations are shown in Table 1. Since the optimal contrast of some coatings increased continuously with the thickness of the coating, we also marked the thickness of 0.95 optimal contrast.

Our simulation results showed that silver had the best contrast-enhancing effect, with a contrast of 13.3185. The optimal contrast of copper significantly increased with wavelength, while the contrast results of chromium-coated tapes were relatively poor.

### 3.2. Imaging Results

**Figure 3:**
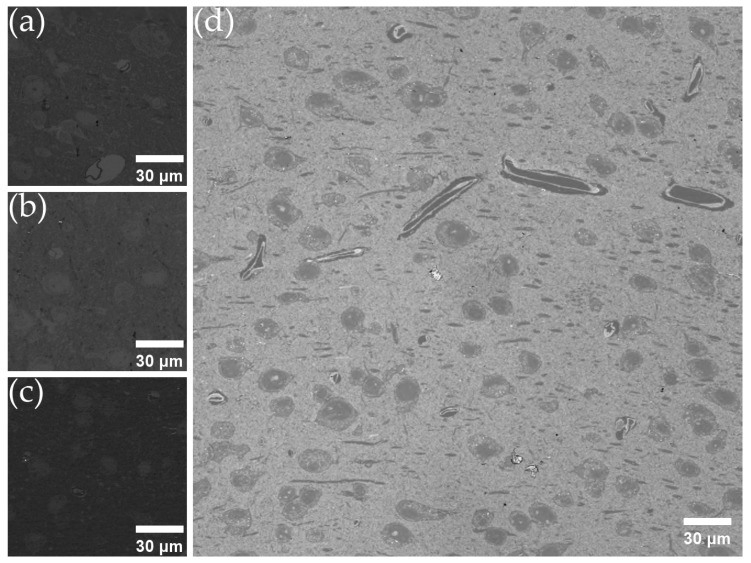
OMLIT imaging results for various combinations of coatings under 470 nm LED illumination with 60 nm thick sections collected on (**a**) 70 nm silver-coated D50 tape; (**b**) 50 nm chromium-coated D50 tape; (**c**) 70 nm copper-coated D50 tape; (**d**) uncoated D50 tape.

We conducted experiments on metal coatings to validate the results of the optical model simulations. However, due to the melting point limitation of the D50 polycarbonate substrate, we could not anneal the metal film layer to make the surface structure flatter. As a result, we observed that the coating was prone to cracking during the winding collection procedure and could be easily oxidized and scratched due to the unprotected surface. In addition to the coated tape, we also used an uncoated tape for collection. The imaging results for the different coatings are presented in Figure 3. Compared to a previous study [46], we found that using monochromatic light as the light source significantly improved the imaging quality under the identical OMLIT configuration.

**Figure 4:**
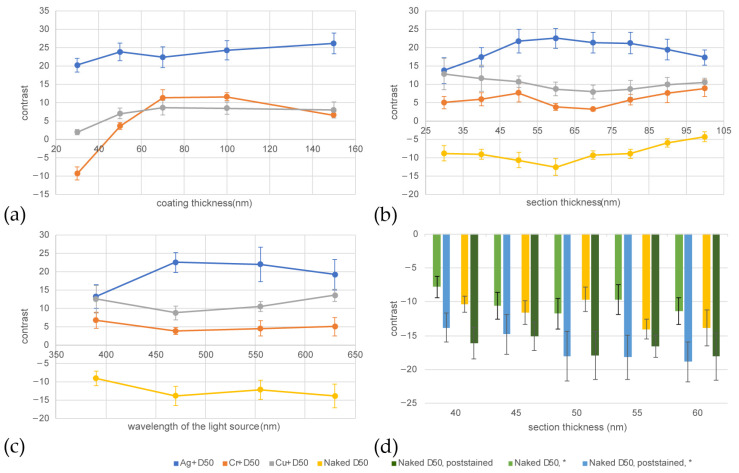
Contrast analysis graphs. (**a**) Under 470 nm illumination, the image contrast obtained from 60 nm thick sample sections collected on D50 tape with varying thicknesses of Ag, Cr, and Cu coatings. (**b**) Under 470 nm illumination, the image contrast obtained from sample sections of various thicknesses collected on D50 tape coated with 70 nm Ag, 50 nm Cr, 70 nm Cu, and naked D50. (**c**) The image contrast obtained from 60 nm thick sample sections collected on D50 tape coated with 70 nm Ag, 50 nm Cr, 70 nm Cu, and naked D50 under various illumination wavelengths. (**d**) Under 470 nm illumination, the comparison of image contrast between poststained and not poststained sample sections of different thicknesses collected on naked D50 tape. To eliminate the randomness of a single sample result, the comparison between poststained and not poststained was repeated with the second mouse cortex sample (*).

We used ImageJ to analyze the contrast of each configuration. The comparison of contrast results for different coating thicknesses, section thicknesses, incident light wavelengths, and with or without poststaining are shown in Figure 4.

We found that the naked D50 tape has a relatively high imaging contrast under monochromatic LED illumination, and it can avoid the adverse effects of the structure of the coating surface, such as scratches and cracks, on the imaging quality. Based on our experimental results, we have confirmed that the poststaining step significantly improves contrast in two different cortex samples. Furthermore, this step can be applied to any metal coating or coating-free method that is not susceptible to displacement reactions with Pb^2+^ ions.

### 3.3. Validation of a Seamless Correlative Light–Electron Hierarchical Imaging Workflow

**Figure 5:**
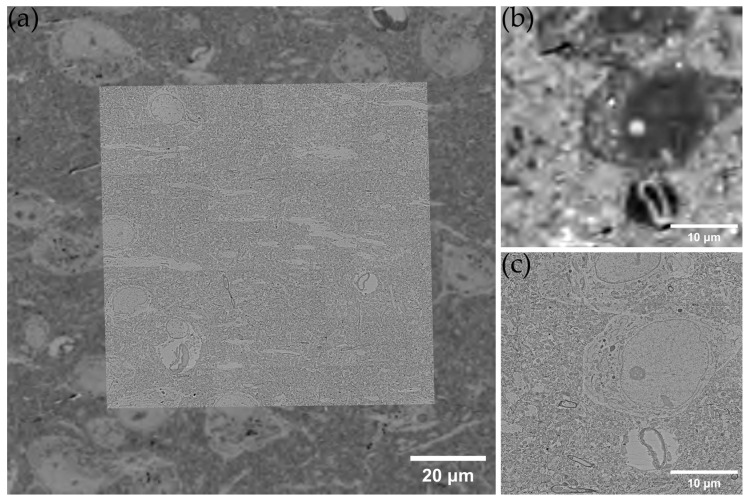
(**a**) The result of co-alignment of light and electron microscopy datasets, in which the optical microscope image is inverted. (**b**) The LM image of a small area with a stretched contrast. (**c**) The EM image of the same area, in which the subcellular organelles can be easily distinguished.

Since the uncoated samples require carbon spraying on the surface to create a conductive layer, we experimented with electron microscopy imaging to determine if this step would interfere with SEM imaging. We found that the EM images of the samples with carbon sprayed on the surface were clear enough to distinguish fine structures, such as nuclear membranes, multilayer myelin sheaths, and cristae of mitochondria (Figure S2). We collected 1657 mouse brain cortex samples with a naked D50 tape and generated an OMLIT dataset (1 × 0.83 × 0.01166 mm^3^) using Olympus VS200. We then performed surface carbon spraying and SEM imaging on this batch of samples to verify the feasibility of this workflow. Additionally, we compared the OMLIT and SEM data of the same section to demonstrate that OMLIT images can guide electron microscopy ROI selection (Figure 5).

Our results showed that optical imaging alone is sufficient to distinguish vasculature, cell bodies, dendrites, and large axons, and is more sensitive to chromatin, which can clearly show the number, size, and distribution of nucleoli in the nucleus that helps determine the cell cycle. Based on electron microscopy images, we presumed a cell to be an astrocyte since they have a large proportion of nucleosomes and a thin layer of heterochromatin distributed beneath the nuclear membrane [55] (pp. 79–83). In the corresponding optical image, we found that the nuclear membrane of this cell was more distinct, consistent, and thicker, as shown in Figure S3.

### 3.4. Large-Volume OMLIT 3D Reconstruction and Biological Analysis

**Figure 6:**
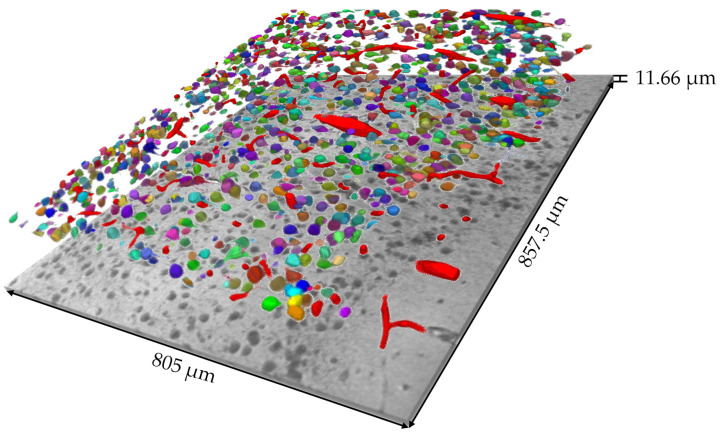
The reconstructed volume and digital volumes of the mouse cortex. A total of 105 unconnected blood vessel sections are highlighted in red, along with 1154 other labeled cell bodies in distinct colors.

In addition to testing the novel scheme of coating-free correlative light–electron hierarchical imaging protocol, we also explored the potential of the OMLIT method to perform large-volume optical reconstructions. We were able to label and trace all blood vessels, cell bodies, nuclei, main dendritic segments, and some larger myelinated axons within the neural tissue at optical resolution. A digital volume of the mouse cortex (350 × 350 × 11.66 μm^3^) was developed as a demonstration (Video S1). Then, 4 experienced annotators labeled the blood vessels and cell bodies and major dendrites and axons in 212 serial optical images (805 × 857.5 × 11.66 μm^3^), shown in Figure 6.

**Figure 7:**
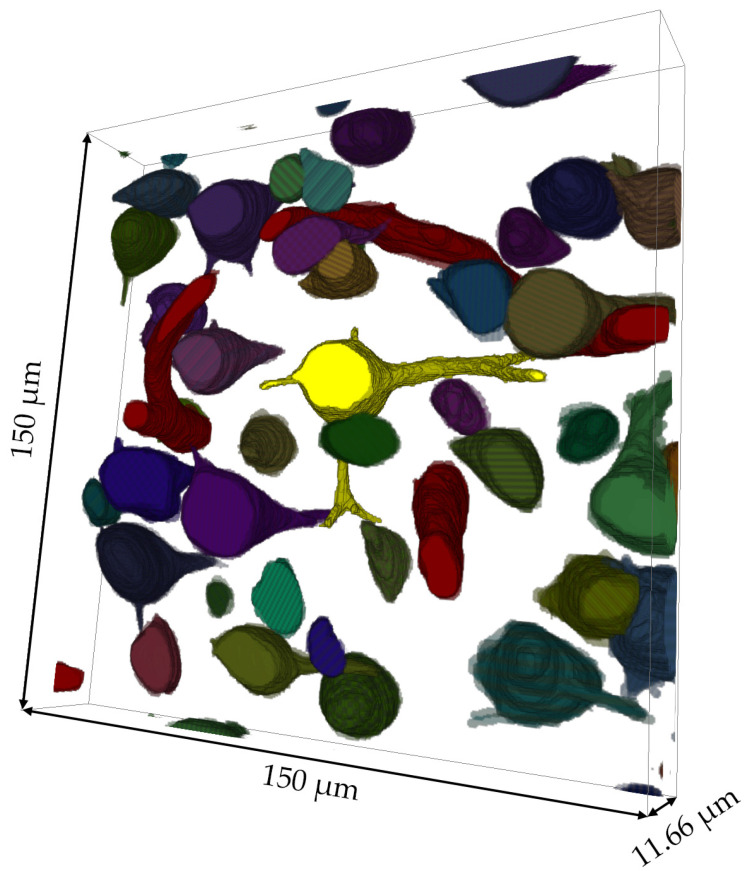
Enlarged view of the reconstructed structures. Highlighted in bright yellow at the center of this reconstructed volume is a nerve cell likely to be a pyramidal cell, with its stout apical dendrite and other neurites clearly distinguishable within the figure.

With the reconstruction volume, we were able to analyze the size and morphology of the neuronal cell bodies and primary dendrites or axons inside this volume. Based on the morphological characteristics of pyramidal cells, we identified the cells with stout apical dendrites and basal dendrites as pyramidal cells [56,57,58,59,60,61]. With a thicker optical reconstruction, preliminary determination of the cell type can be made based on the volume of the cell body. Our method can aid in cell classification, cell density, and volume analysis. Figure 7 and Video S2 illustrate a cell that is most likely a pyramidal cell and the direction of the main segments of the neurites of this cell. The stout apical dendrite, the basal dendrites, and a small segment of the axon of this cell can all be observed with our method. After identifying cells with large apical dendrites and conical cell bodies in the entire volume of this sample, we marked them in light blue (Figure S4) and conducted a statistical analysis. The results showed that these large-likelihood pyramidal cells occupied 28.7% of the entire volume of 1154 cells [62,63]. The relationship between the reconstructed volume and cell volume influences the low ratio of likely pyramidal cells, particularly with larger cell volumes. This results in the likely pyramidal cells being more prone to appearing at the edges of the reconstructed volume and not being discerned.

### 3.5. Thicker Section Imaging Results

**Figure 8:**
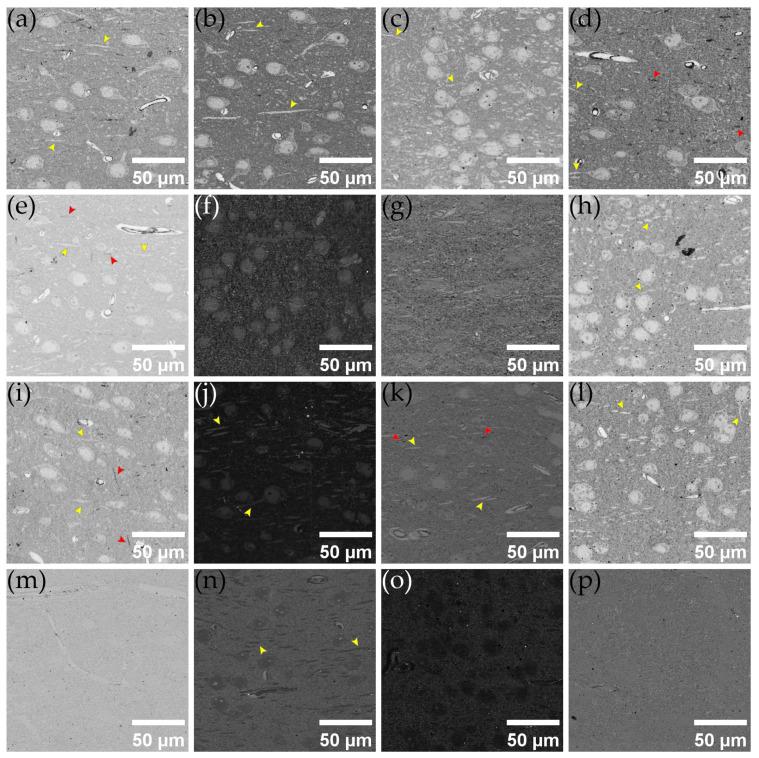
OMLIT imaging results for thicker sample sections under 470 nm LED illumination. Sections of 150, 200, 250, and 300 nm samples collected on (**a**–**d**) 70 nm silver-coated D50 tape; (**e**–**h**) 50 nm chromium-coated D50 tape; (**i**–**l**) 70 nm copper-coated D50 tape; (**m**–**p**) uncoated D50 tape. In some of the subfigures, yellow arrows are used to indicate dendritic components, while red arrows are employed to point out axonal components.

In the previously developed OMLIT protocol, ultra-thin sections of tens of nanometer thickness gives a vertical resolution that is much higher than the lateral resolution of optical imaging. This leads to significant inefficiencies in imaging time, as well as effective data size, when attempting to perform a rapid optical reconstruction process. Choosing to image at intervals of several sections can reduce data occupancy but still cannot avoid unnecessary expenditure of collection time and costs. To tackle this issue, we first extended the optical model to apply to much thicker sections. Simulations showed that the image contrast actually periodically fluctuated and was relatively good even at thicknesses close to visible light wavelengths. Thus, we collected thicker sections to observe the efficacy of OMLIT imaging, and the results are displayed in Figure 8.

Remarkably, even on sections hundreds of nanometers thick, OMLIT images were sufficient to distinguish blood vessels, cell bodies, nuclei, dendrites, and axons. We found that, among the test results for several thicker sections, the silver coating still provided the best contrast, while the coating-free method that works so well in thin sections may no longer be feasible. For a comprehensive analysis of contrast data from thicker sections, please refer to Table S1.

## 4. Discussion

In this study, we explored various coating schemes of OMLIT and tested several metals with excellent ductility and electrical conductivity. By combining different configurations of physical parameters, such as refractive index and extinction coefficient, we conducted simulations of the imaging contrast of various metals. The experiments show a trend consistent with the simulated results. Specifically, we propose one of the most convenient and ATUM-SEM-correlated imaging protocols in existence, which is well suited for the majority of laboratory facilities. The method requires only a small commercial carbon coating machine. We built a demonstration of this scheme on a relatively large cortex volume, obtaining mesoscale and microscale images of the same mouse cortex sample. We also expanded the range of sample thicknesses.

Based on the results of the optical simulations, we found that the imaging contrast of the OMLIT method fluctuates with increasing thickness of the sample sections. The period of contrast fluctuation is positively correlated with the incident light wavelength. This phenomenon occurs because the image contrast is generated by the interference between the light reflected from different film interfaces. By adjusting the incident light wavelength, our method can obtain an improvement in imaging quality on thicker sections, which was confirmed in the experiment. The method was shown to be able to improve optical image contrast even at sample section thicknesses of hundreds of nanometers, close to visible light wavelength and lateral optical resolution. This means that our method can be used to improve the contrast of samples ten times thicker than the previously reported OMLIT and save significant imaging time as a fast and efficient stand-alone all-cell optical imaging method.

In the previous study of OMLIT, the impact of the multilayer optical film structure on imaging contrast was evaluated under white LED light sources. In our study, we used the same OMLIT configuration and achieved better results due to the use of a monochromatic LED light source. The improved contrast can be attributed to the reduced aberrations (including the chromatic aberration, the spherical aberration, etc.) of the brightfield microscope when using a monochromatic LED source compared to the one using a white LED light source. Because the monochromatic LED light source is a low-coherence source compared with its laser counterpart, the speckles in the final images are reduced, leading to a much higher image quality and contrast [64,65,66]. Our results indicate that the performance of monochromatic LED light sources is superior to that of white LEDs when employing the OMLIT method. We should emphasize again that this method is designed for low-coherence sources and not intended for laser imaging. This is because the sample in our case consisted of randomly distributed materials inside, which indicates a strong speckle and low image contrast when using a high-coherence laser as the imaging source.

Our results are consistent with the previous study of OMLIT, showing that coatings of substrate tapes are troubled with background noise to varying extents. This generally comes from the unevenness of the film surface, such as fiber structures of commercially coated carbon nanotube polyethylene terephthalate (PET) tape, scratches, cracks, and peeling of the metal layer of the metal-coated Kapton or Panlite tapes. Since the imaging effect of the OMLIT method is mainly determined by the interference of the multilayer film structure, these film surface structures can then seriously affect the imaging quality and randomly obscure the region of interest, resulting in data loss. The experiments in this study suggest that these background noise interferences are mostly eliminated in the coating-free OMLIT method. In other words, the coating-free method can minimize the noise caused by structural defects on the film surface and thus improve the imaging results.

Despite the significant achievements in this study, there are still some limitations that need to be addressed in future research. Firstly, the number of coating materials tested in this study was relatively small. A more extensive exploration of coating materials and combinations could potentially lead to the identification of even better coating schemes, improving image quality and reducing artifacts. Systematic investigations into these coatings, with a focus on their optical and chemical properties, could provide valuable insights into optimizing the imaging process. Secondly, the current optical model agrees with experimental results only in terms of contrast trend, while the peak position and contrast value may deviate from observed data. This discrepancy might be attributed to inaccuracies in the refractive index and extinction coefficient of the relevant materials. To overcome this limitation, techniques such as ellipsometry should be applied to obtain accurate optical parameters of these materials at specific thicknesses. The refinement of the optical model would enable better predictions and potentially pave the way for further optimization of the imaging process.

In addition to addressing these limitations, there are several promising future directions worth investigating. The datasets generated by this study have been utilized in research involving automatic segmentation algorithms and super-resolution algorithms with correlated images of OMLIT and serial SEM. With the help of machine learning, we hope to enhance the imaging throughput of our method and potentially improve its resolution through algorithmic advancements. Furthermore, the development of more sophisticated algorithms could facilitate the identification of subtle structural features and enable more accurate quantitative analysis of neural tissue. Moreover, integrating our method with molecular labeling techniques [67,68,69,70,71] resistant to osmium staining and resin embedding could broaden its applications in neuroscience. This integration would enable all-cell imaging of both fine structures and molecular imprints of biological tissues, providing a more comprehensive understanding of cellular and molecular processes within the nervous system. It could also facilitate the study of brain disorders at a molecular level, potentially revealing novel targets for therapeutic intervention. Lastly, correlating our method with functional studies, such as electro- and magnetoencephalography (EEG and MEG) [72,73], represents a valuable future direction. These techniques offer insights into various aspects of the brain’s structural and functional architecture. By combining our approach with these methods, we could create a comprehensive multimodal perspective of the central nervous system, enhancing our understanding of brain functionality and enabling more accurate diagnosis of brain disorders. This integration could lead to a more holistic view of the brain, bridging the gap between structure and function, and ultimately advancing our knowledge of how this high-performance imaging method could contribute to diagnosing and treating various brain disorders.

## 5. Conclusions

In conclusion, we have developed an optimized coating-free optical multilayer interferometric imaging method that offers a high level of convenience and compatibility with ATUM-SEM for mesoscale and microscale brain mapping. Our method is accessible to most laboratory facilities and requires only a small commercial carbon-coating instrument. We demonstrated the effectiveness of this approach by imaging a large volume of mouse cortex and verifying seamless compatibility with multibeam SEM. Despite some limitations, the optimized OMLIT method shows great promise for improving the efficiency and automation of large-scale merged brain mapping. Future research should focus on addressing these limitations and further enhancing the capabilities of this innovative imaging approach.

In terms of limitations and future directions, several aspects of our study warrant further investigation. Firstly, we need to explore a wider range of coating materials and combinations to potentially identify more optimal coating schemes. Secondly, the accuracy of the current optical model should be improved by obtaining precise optical parameters for the relevant materials using techniques such as ellipsometry. Additionally, we propose using artificially segmented datasets to train deep learning algorithms, which can automatically segment structures such as cell bodies, primary dendritic initiation segments, and capillaries. In combination with faster mesoscale and microscale imaging methods and thicker sample sections, this approach could lead to large-scale, merged brain mapping methods with increased throughput and automation. Furthermore, integrating our OMLIT method with molecular labeling techniques resistant to osmium staining and resin embedding could broaden its applications in neuroscience research. This would involve combining high-resolution imaging of fine structures and molecular imprints of biological tissues. Lastly, future work could explore the possibility of correlating our method with functional studies, such as EEG and MEG. This integration could create multimodal brain maps, enhancing brain function analysis and disease diagnosis, leading to a more comprehensive understanding of brain complexity. By addressing these limitations and pursuing these future directions, we can further advance the field of brain mapping and contribute to a deeper understanding of the nervous system.

## Figures and Tables

**Table 1:** Predictions of the optimal contrast in different combinations of coating material, thickness, and slice thickness on D50 polycarbonate film substrates according to the OMLIT model. The units of wavelength, tissue thickness, and coating thickness are in nm.

Coating Material	Best Contrast	Wavelength	Tissue Thickness	Coating Thickness
Chromium	2.4883	390	40	20
	1.8434	470	150	50
	1.802	555	190	50
	2.1049	630	70	40
Copper	2.5216	390	30	30
	2.5058	470	30	110
	5.3257	555	40	180
	11.5284	630	60	400 (80) *
Silver	13.3185	390	20	200
	12.3006	470	40	400 (70) *
	12.5592	555	50	400 (50) *
	12.0547	630	60	400 (70) *

* The thickness of the coating corresponding to the 95% optimum contrast is shown in parentheses.

## Data Availability

The data presented in this study are openly available in FigShare at https://doi.org/10.6084/m9.figshare.22186675.v1, accessed on 28 February 2023.

## Supplementary Materials

The following supporting information can be downloaded at: https://www.mdpi.com/article/10.3390/brainsci13050711/s1, Figure S1: Contrast simulation result for chromium coating; Figure S2: SEM image of the surface carbon-coated sample; Figure S3: Light and electron microscopic images of a cell with a thin layer of heterochromatin attached to the nuclear membrane; Figure S4: Identification and quantification of large-likelihood pyramidal cells; Table S1: Contrast of thicker sections; Video S1: Animation of vasculature and neurite extensions in the OMLIT dataset; Video S2: An animation showcasing the reconstructed morphology of a nerve cell, likely to be a pyramidal cell.
