# Supplementary material for: A Convenient All-Cell Optical Imaging Method Compatible with Serial SEM for Brain Mapping
Source: Brain Sci. 2023 Apr 24;13(5):711. doi: 10.3390/brainsci13050711 (PMC10216590; doi:10.3390/brainsci13050711)
Supplement: Supplementary file 1 [file brainsci-13-00711-s001.zip › Supplementary Materials.pdf]

Supplementary Materials: A convenient all-cell optical imaging method compatible with serial SEM for brain mapping

Tianyi Wang [1,2], Peiyao Shi [2], Dingsan Luo [2], Jun Guo [3], Hui Liu [2], Jinyun Yuan [2], Haiqun Jin [3], Xiaolong Wu [3], Yueyi Zhang [3], Zhiwei Xiong [3], Jinlong Zhu [4], Renjie Zhou [5], and Ruobing Zhang [1,2,3] *

[1] School of Biomedical Engineering (Suzhou), Division of Life Sciences and Medicine, University of Science and Technology of China, Suzhou 215163, China
[2] Jiangsu Key Laboratory of Medical Optics, Suzhou Institute of Biomedical Engineering and Technology, Chinese Academy of Science, Suzhou 215163, China
[3] Institute of Artificial Intelligence, Hefei Comprehensive National Science Center, Hefei 230088, China
[4] State Key Laboratory of Digital Manufacturing Equipment and Technology, Huazhong University of Science and Technology, Wuhan 430074, China
[5] Department of Biomedical Engineering, The Chinese University of Hong Kong, Hong Kong 999077, China
* Correspondence: zhangrb@sibet.ac.cn

List	*Page*
Authors	S1
Manuscript Title	S1
Figure S1	S2
Figure S2	S2
Figure S3	S3
Figure S4	S3
Table S1	S4
Video S1	S4
Video S2	S4

Figure S1. Contrast simulation result for chromium coating. The red dots denote the locations of maximal contrast, with corresponding contrast conditions indicated in the legends.

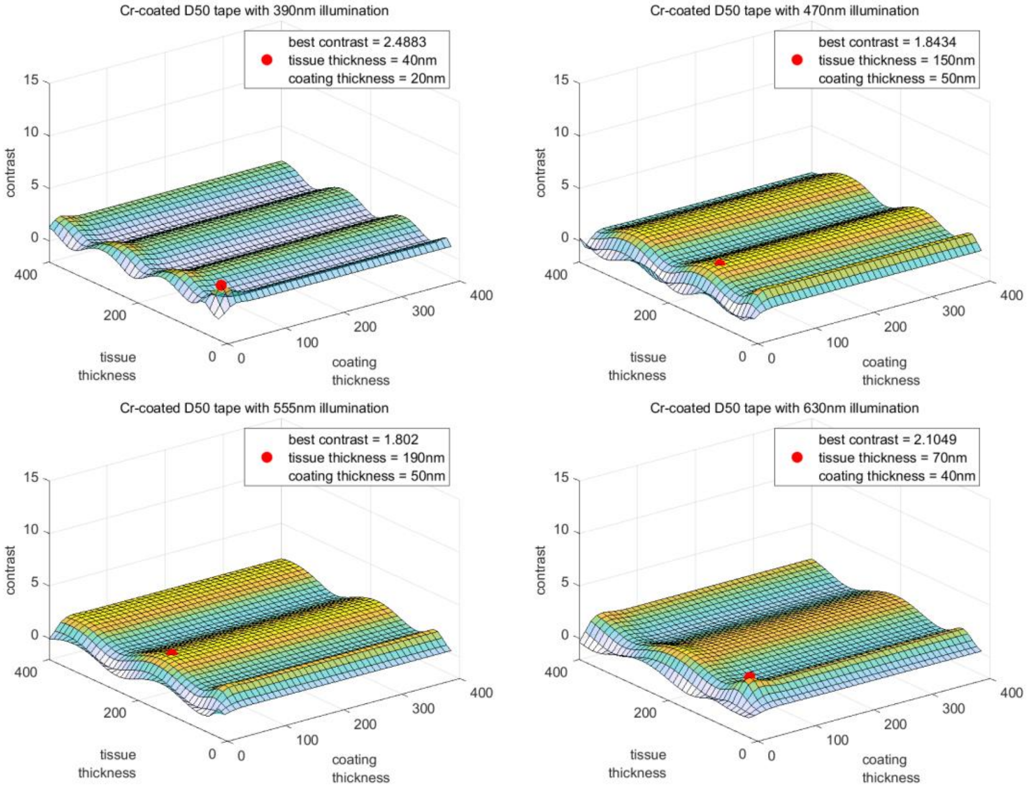

Figure S2. SEM image of the surface carbon-coated sample. Fine structures such as nuclear membranes, multilayer myelin sheaths, and cristae of mitochondria can be distinguished.

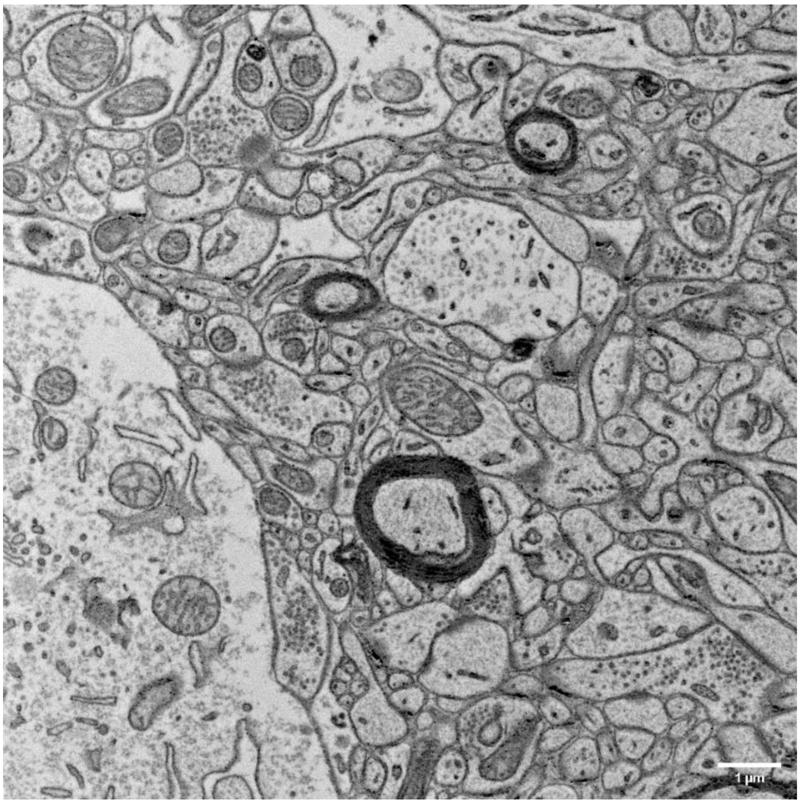

Figure S3. Light and electron microscopic images of a cell with a thin layer of heterochromatin attached to the nuclear membrane.

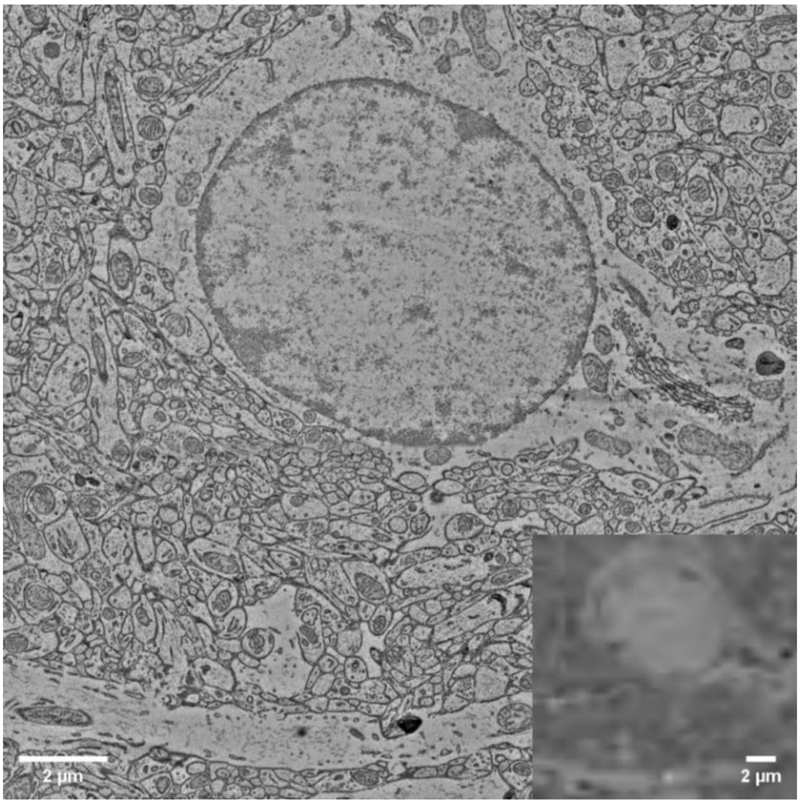

Figure S4. Identification and quantification of large-likelihood pyramidal cells. Cells with large apical dendrites and conical cell bodies were marked in light blue.

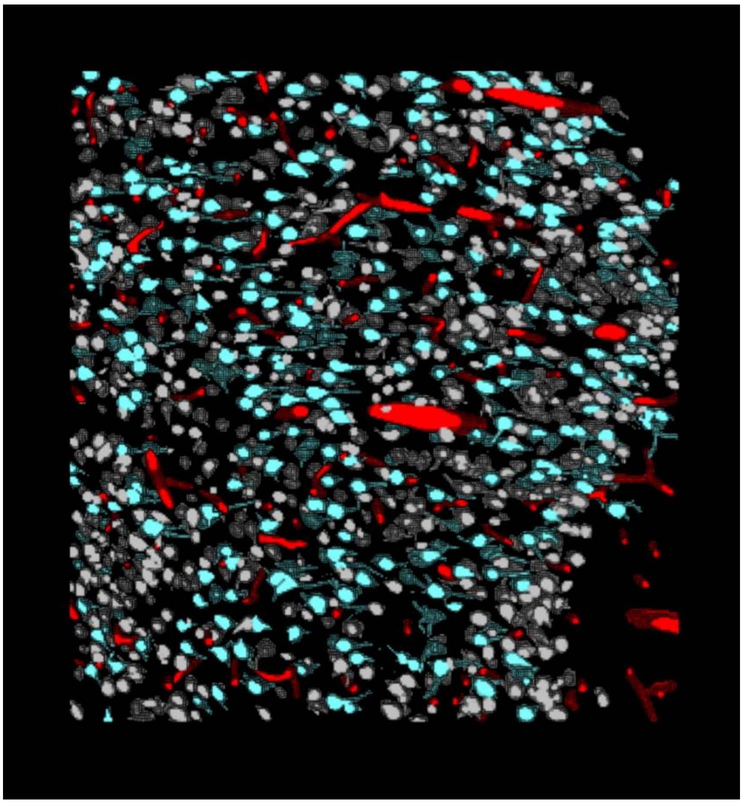

Table S1. Contrast of thicker sections.

Coating material	Tissue thickness			
	**150 nm**	**200 nm**	**250 nm**	**300 nm**
Chromium	11.7189 ± 1.401	5.7336 ± 1.586	5.343 ± 2.7227	16.4423 ± 2.1038
Copper	16.2642 ± 3.034	10.1775 ± 1.5299	13.6859 ± 2.2995	28.9886 ± 3.7851
Silver	24.9702 ± 4.313	43.1763 ± 4.517	29.2957 ± 2.9186	45.6054 ± 6.0187
-	1.6012 ± 0.87409	-5.9495 ± 1.8505	-3.0735 ± 0.81519	1.6427 ± 1.3599

Video S1. The animation of vasculature and neurite extensions in the OMLIT dataset.

Video S2. An animation showcasing the reconstructed morphology of a nerve cell, likely to be a pyramidal cell.
